# Detection of a Putative Novel Papillomavirus Type within a Large Exophytic Papilloma on the Fetlock of a Horse

**DOI:** 10.3390/pathogens9100816

**Published:** 2020-10-05

**Authors:** John S. Munday, Michael R. Hardcastle, Melissa Sim

**Affiliations:** 1 School of Veterinary Science, Massey University, Palmerston North 4410, New Zealand; 2 Gribbles Veterinary Pathology Ltd., Auckland 1060, New Zealand; Michael.Hardcastle@gribbles.co.nz; 3 Franklin Vets, Pukekohe 2120, New Zealand; msim@fvs.co.nz

**Keywords:** horse, papillomavirus, papilloma, *Zetapapillomavirus*, wart, skin

## Abstract

A 10-year-old horse presented with two 3-cm diameter exophytic masses over the fetlock. Histology was consistent with a hyperplastic squamous papilloma and numerous cell changes consistent with papillomavirus (PV) infection were visible. Partial sequences of PV L1 and E1 ORFs were amplified using consensus PCR primers. The sequences were most similar to Equus caballus type 1 (EcPV1). However, as the sequences were only around 73% similar to EcPV1, they appear to be from a novel PV type that is likely to be within the *Zetapapillomavirus* genus. The papillomas were treated with topical imiquimod and resolved within 14 weeks. The clinical presentation of the papillomas in the present case had marked differences to the clinical presentation of EcPV-1-induced papillomas, which are typically small, numerous and around the face. Observations from the present case increase the clinical spectrum of PV-induced lesions in this species as well as providing evidence of an additional novel papillomavirus that is able to cause disease in horses.

## 1. Introduction

Papillomaviruses (PVs) are small circular double-stranded DNA viruses that generally infect epithelium and are typically highly host specific [1]. Numerous cutaneous and mucosal PV types have been found to infect humans and papillomaviruses have been detected in almost all non-human species that have been studied [1,2]. The majority of PV infections are asymptomatic [2]. However, a subset of PV types can cause self-resolving hyperplastic lesions (warts). Furthermore, some human PV types have been shown to cause cancer, with an increasing number of neoplasms in animals also associated with PV infection [3,4,5]. 

Horses are well recognized to develop disease due to PV infection and nine equine papillomaviruses have currently been fully sequenced and deposited in GenBank. Equus caballus type 1 (EcPV1) was the first PV type fully sequenced from horses and this *Zetapapillomavirus* causes papillomas in horses [6]. Infection by EcPV-1 is thought to be by direct contact or through infected fomites and horses typically develop 10–100, 0.2–2 cm diameter pedunculated lesions that are most common on the head, especially the muzzle, eyelids and lips. Lesions self-resolve after 4–9 months and horses do not develop further papillomas [7]. Genital papillomas have been associated with EcPV2, a *Dyoiotapapillomavirus* and EcPV7, a *Dyorhopapillomavirus* [4,5,8]. Recent evidence suggests that EcPV2 may also have a causative role in a proportion of equine genital, oral, and gastric cancers [9,10]. The method of transmission of EcPV2 is currently unknown. Equus caballus types 3, 4, 5, and 6 have all been associated with aural plaques [8,11]. These plaques develop on the concave surface of the ear and tend to persist. The causative PVs are thought to be spread by biting flies and lesions are restricted to geographical locations in which these flies are present [12]. Both EcPV3 and 6 are *Dyorhopapillomaviruses* while EcPV4 and 5 are classified as *Dyoiotapapillomaviruses* [8,11]. Recently, EcPV8 was reported in association with the development of massive numbers of cutaneous papillomas covering a wide area of the skin [13]. As EcPV9 was detected in semen from a clinically normal horse, whether this PV causes disease is currently uncertain [14]. In addition to diseases caused by the equine PV types, horses also commonly develop sarcoids due to cross-species infection by bovine *Deltapapillomavirus* types [2]. 

Herein is reported a horse that developed cutaneous papillomas that showed marked clinical differences from the typical cutaneous papillomas that develop in horses. A DNA sequence that is most likely to be from a previously undescribed PV type was amplified from the lesion. 

## 2. Case Presentation and Diagnosis

**Figure 1:**
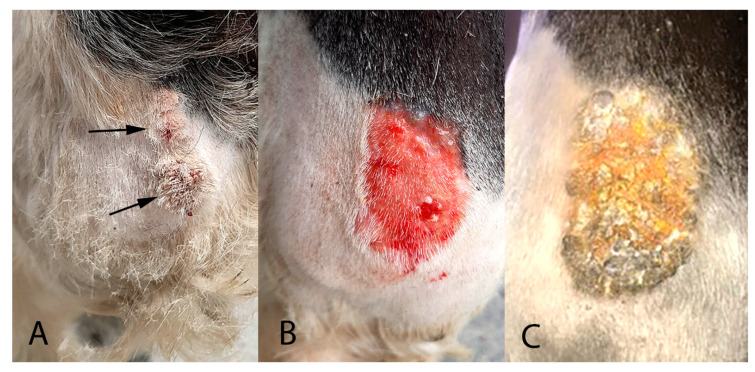
(**A**). The horse presented with two approximately 3 cm diameter exophytic masses over the left front fetlock (arrows). The masses had a hard, roughened surface. (**B**). The area was debrided using a saline swab after daily topical application of 2 sachets of 5% imiquimod cream (Aldara, iNova Pharmaceuticals (Australia) Pty, Chatswood, Australia) for 10 days. While the area is markedly reddened, only mild thickening of the skin is present. (**C**). After treatment for two additional weeks using 1 sachet of 5% imiquimod cream applied every 72 h, the area is mildly reddened and multifocally covered with thick serocellular crusts. However, no thickening of the underlying skin was detectible at this stage.

A 10-year-old warmblood gelding presented with two exophytic roughened 3 cm-diameter masses on the cranial aspect of the left front fetlock (Figure 1A). 

The masses had been first noticed three weeks previously and had grown rapidly since this time. One of the masses was partially excised, fixed in formalin, and submitted for routine histological evaluation at a diagnostic laboratory. 

**Figure 2:**
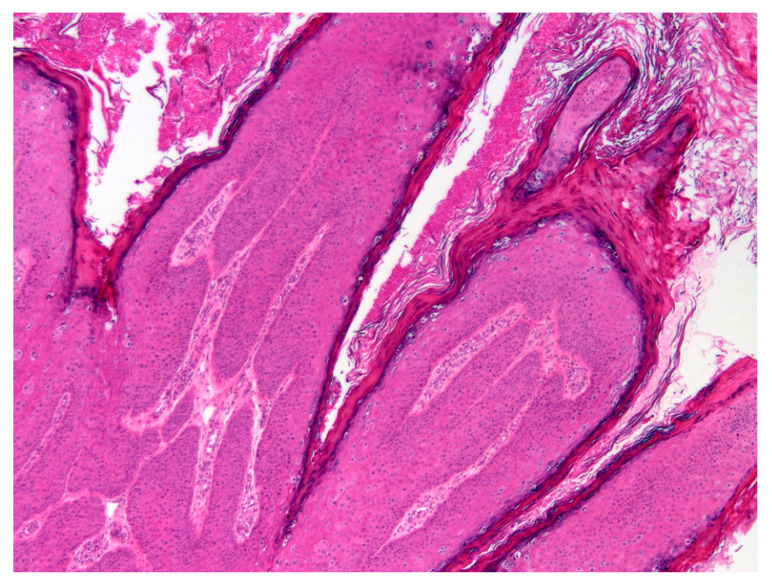
Photomicrograph of the mass from the fetlock. The mass consists of thickened epidermis that has become folded, resulting in filiform projections supported by minimal fibrovascular stroma. H&E 100×.

Histology revealed an exophytic mass that consisted of markedly thickened epidermis arranged in filiform projections supported by a minimal fibrovascular core (Figure 2). 

**Figure 3:**
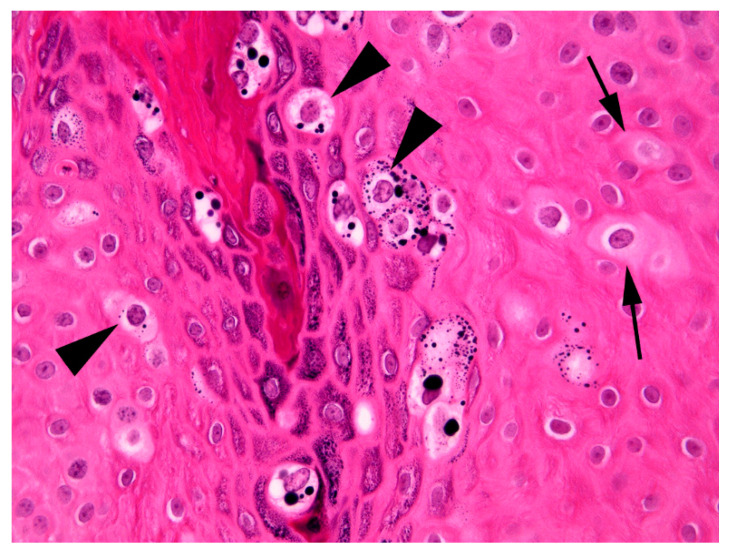
Photomicrograph of the mass from the fetlock. Enlarged cells that have increased quantities of blue-grey cytoplasm are visible within the thickened epidermis (arrows). Additionally, enlarged cells that have shrunken nuclei that are surrounded by a clear cytoplasmic halo (koilocytes; arrowheads) are also visible. Note also the prominent clumping of keratohyalin granules superficially within the lesion. H&E 400×.

The thickening was well-demarcated and surrounding epidermis appeared within normal limits. Examination of the thickened epidermis revealed that orderly maturation was maintained and the proliferative lesion was covered by marked dense orthokeratosis. There was no evidence of invasion of the basement membrane and few inflammatory cells were visible within the superficial dermis or within the deeper layers of the hyperplastic epidermis. Scattered within the superficial aspects of the stratum spinosum and the stratus granulosum were numerous keratinocytes that were enlarged by increased quantities of pale blue cytoplasm (Figure 3).

Cells within the superficial epidermis that showed this change also often contained large numbers of clumped keratohyalin granules. In addition to the large cells with blue-grey cytoplasm there were also smaller numbers of cells that had a shrunken nucleus surrounded by a clear halo (koilocytes). The enlarged cells with blue-grey cytoplasm and koilocytosis were considered consistent with PV-induced cell changes and a diagnosis of a viral squamous papilloma (wart) was made. 

DNA was extracted from a formalin-fixed paraffin-embedded tissue scroll of the lesion using a High Pure PCR Template Preparation kit (Roche Diagnostics GmbH, Mannheim, Germany) according to the manufacturer’s instructions. Primers used to amplify DNA included the consensus primers FAP59/64, which were designed to amplify a variety of human cutaneous PV types, MY09/11, which were designed to amplify a variety of human mucosal PV types, and CP4/5 that amplify the E1 ORF of a variety of human and non-human PV types. DNA was extracted from an equine penile papilloma that contained EcPV2 DNA as a positive control for the FAP59/64 and MY09/11 primers and DNA was extracted from a canine oral papilloma that contained Canis familiaris PV1 DNA for the CP4/5 primers [15]. No template DNA was added to the negative controls. Amplified DNA was sequenced as previously described [15] and the NCBI Blast tool was used to compare the sequences to those previously deposited in GenBank. 

Papillomaviral DNA was amplified from the mass using the MY09/11 and CP4/5 primers. Sequencing of the MY09/11 amplicon produced a 405 bp section of the PV L1 ORF. A comparison of this sequence to the PV sequences previously deposited in GenBank revealed the closest similarity to EcPV1. However, the new sequence was only 73.5% similar over the 405 bp region to both the initially reported EcPV1 sequence (GenBank AF498323) [6] and a recently reported variant of EcPV1 that was amplified from a papilloma on the face of a racehorse in Japan (GenBank MF288893) [16]. A comparison with other EcPV types revealed 58.1% similarity to EcPV2, 60.1% similarity to EcPV7, 54% similarity to EcPV8 and 56.4% similarity to EcPV9. The novel sequence amplified from the papilloma was deposited into GenBank (accession number MT947747). Sequencing of the CP4/5 product also revealed the highest similarity to EcPV1, with around 75% similarity between the novel sequence and EcPV1. No PV DNA was amplified by the FAP59/64 primers, although DNA was amplified from the positive control as expected. The extraction, amplification and sequencing reactions were repeated and this confirmed the initial findings.

The viral papillomas were treated by daily topical application of 2 sachets of 5% imiquimod cream (Aldara, iNova Pharmaceuticals (Australia) Pty, Chatswood, Australia) for 10 days. After this treatment, the papillomas were reduced in size and covered by a thick serocellular crust. The crust was debrided using a saline swab (Figure 1B) and samples of the crust were evaluated for the presence of PV DNA using the previously described methods. However, no PV sequences could be detected in samples of crust taken at this time. 

Some residual thickening of the epidermis was present and the horse was treated for a further two weeks using 1 sachet of 5% imiquimod cream applied every 72 h. At this time, the skin was observed to be mildly reddened and multifocally covered with a thick serocellular crust (Figure 1C). A swab of the area was taken, which again did not contain amplifiable PV DNA sequences. No significant thickening of the underlying skin could be detected at this time. 

Four months after the papillomas were first observed and ten weeks after the completion of the treatment, clinical examination of the area of skin did not reveal any significant abnormalities.

## 3. Discussion 

A variety of proliferative skin lesions have been reported in horses due to infection by equine papillomaviruses. Papillomas due to EcPV1 are most common and these papillomas are typically small, numerous and develop around the head [7]. Papillomas due to EcPV2 and 7 are restricted to the genitals while the papillomas that have been reported due to EcPV8 are small and numerous over a wide area of the body [7,13]. Aural plaques appear as sessile masses that are restricted to the ears and are associated with EcPV3, 4, 5, and 6 [8,11]. In contrast to the previous descriptions of EcPV-associated skin disease in horses, the presently described horse developed two large papillomas over the fetlock. Due to their size and location, these papillomas were initially thought to be atypical sarcoids. The unusual clinical presentation in this horse increases the number of clinical manifestations of PV skin disease in horses and infection by these viruses should be considered when small numbers of large skin masses develop in a horse. 

In addition to their unusual clinical presentation, the papillomas in the present case may also have been associated with a novel PV type. Papillomaviruses are classified using the L1 ORF with papillomaviruses with less than 90% similarity considered to be a different type and those with less than 60% considered to be different genera [1]. As only a partial sequence of the L1 gene was amplified in the present case, definitive classification is not possible. However, as the partial sequence was only around 73% similar to EcPV1, this suggests the virus is likely to be an additional, previously undetected EcPV type. The level of similarity to EcPV1 suggests that it is likely the novel PV type will also be classified within the *Zatapapillomavirus* genus. Papillomaviruses within the same genus often cause similar lesions [1]. The observation that both EcPV1 and the presently reported PV type appear to cause squamous papillomas of the skin increases the likelihood that the putative novel PV type will be within the same genus as EcPV1. 

As infection by PVs is often asymptomatic [17], simply detecting a PV within a lesion does not prove causality. However, many of the histological features in the present case are considered to be characteristic of PV-induced papillomas in horses as well as in other species [18]. These features include the presence of folded epidermis due to an area of well-demarcated epidermal hyperplasia. This change occurs as the PV infection stimulates expansion of the epidermis. Importantly, as observed in the present case, the PV does not alter the normal maturation of the epidermis as would be seen in pre-neoplastic or neoplastic epidermal lesions. Additionally, PV-induced cytopathic changes were visible in the hyperplastic epidermis. These changes included the presence of enlarged cells with blue-grey cytoplasm, clumping of keratohyalin granules, and koilocytosis [18]. 

Histology provided strong evidence that the papillomas were caused by PV infection. However, studies have shown bovine papillomas often contain multiple different PV types [19]. Therefore, it is more difficult to prove that the papillomas were caused by the putative novel PV type and not by a different, undetected PV type within the lesion. However, as the FAP59/64 primers have been previously used to amplify EcPV2 and EcPV8 DNA [4,13], the failure of these primers to amplify DNA from the papilloma in the present case confirms that neither of these PV types were present. Additionally, marked epidermal hyperplasia has not been reported due to EcPV3, 4, 5, 6, 7, or 9, suggesting these types are unlikely to have caused the viral papillomas observed in the present case. Co-infection by EcPV1 is difficult to exclude, although the clinical appearance of the warts in the present case would be considered unusual for papillomas caused by this PV type.

Infection by a PV occurs when microtrauma allows contact between a PV virion and the basal cells of the epidermis [20]. As the feet and extremities are often traumatized, warts are common in this location in many species [3]. While there was no history of trauma in this case, it appears likely that mild trauma to the fetlock allowed entry of the virus and subsequent papilloma development. The source of the PV infection in the present case is uncertain. The horse attended a show approximately 8 weeks prior to the development of the lesions and infection due to direct contact with an infected horse appears most likely. However, the possibility that the horse was indirectly exposed to the PV from contaminated tack or even veterinary equipment cannot be excluded.

Papillomas only develop when a host is infected by a PV type for the first time [21]. As most horses are infected by EcPV1 early in life, warts caused by this PV type typically develop in young horses [7]. In contrast, the presently described horse was 10-years-old when the papillomas developed. This suggests that the horse was not exposed to the putative novel PV type until later in life and therefore this PV type may be less prevalent than EcPV1. If infection by the putative novel PV type is rare in horses, this would explain why this PV type appears to have remained undetected until now. 

The presently described horse was treated with imiquimod cream. As virally induced squamous papillomas are expected to self-resolve, the degree to which the treatment influenced lesion resolution is unknown. The effect of the treatment is especially difficult to determine in the present case as it is possible that papillomas associated with the putative novel PV type have a different clinical behaviour to papillomas caused by EcPV1. Imiquimod stimulates an inflammatory response against the epithelium [22]. While it was initially used to treat human genital warts, imiquimod does not have any specific action against PVs and is now used to treat some non-viral skin cancers in humans [23]. The use of the cream in the present case was associated with significant ulceration and reddening of the skin. This is thought to be due to the stimulation of local inflammation and is a well-recognized side effect in people using this topical treatment [23]. 

## 4. Conclusions

The clinical presentation of the papillomas in the present case was unusual and unlike that typically observed in horses with papillomas that have been caused by EcPV1. While the histology of the lesion was typical for a PV-induced papilloma, the papilloma contained DNA sequences that were most likely from a novel PV type. This suggests additional PV types are able to infect horses. Additionally, this report adds to the clinical manifestations of PV disease in this species.
